# Interaction between Marine-Derived *n*-3 Long Chain Polyunsaturated Fatty Acids and Uric Acid on Glucose Metabolism and Risk of Type 2 Diabetes Mellitus: A Case-Control Study

**DOI:** 10.3390/md13095564

**Published:** 2015-08-26

**Authors:** Kelei Li, Kejian Wu, Yimin Zhao, Tao Huang, Dajun Lou, Xiaomei Yu, Duo Li

**Affiliations:** 1 Department of Food Science and Nutrition, Zhejiang University, Hangzhou 310058, China; E-Mails: wfujfqcc@sina.com (K.L.); wkj016b@163.com (K.W.); davidzhao2147@gmail.com (Y.Z.); 2 Department of Nutrition and Epidemiology, Harvard School of Public Health, Boston, MA 02115, USA; E-Mail: taohuang83@gmail.com; 3 Department of Endocrinology, Shaoxing Hospital, Shaoxing 312000, China; E-Mail: loudajun@sina.com; 4 Department of Clinical Laboratory, Zhejiang Hospital, Hangzhou 310013, China; E-Mail: yuxiaomei_1@hotmail.com

**Keywords:** *n*-3 polyunsaturated fatty acids, uric acid, type 2 diabetes, human

## Abstract

The present case-control study explored the interaction between marine-derived *n*-3 long chain polyunsaturated fatty acids (*n*-3 LC PUFAs) and uric acid (UA) on glucose metabolism and risk of type 2 diabetes mellitus (T2DM). Two hundred and eleven healthy subjects in control group and 268 T2DM subjects in case group were included. Plasma phospholipid (PL) fatty acids and biochemical parameters were detected by standard methods. Plasma PL C22:6*n*-3 was significantly lower in case group than in control group, and was negatively correlated with fasting glucose (*r* = −0.177, *p* < 0.001). Higher plasma PL C22:6*n*-3 was associated with lower risk of T2DM, and the OR was 0.32 (95% confidence interval (CI), 0.12 to 0.80; *p* = 0.016) for per unit increase of C22:6*n*-3. UA was significantly lower in case group than in control group. UA was positively correlated with fasting glucose in healthy subjects, but this correlation became negative in T2DM subjects. A significant interaction was observed between C22:6*n*-3 and UA on fasting glucose (*p* for interaction = 0.005): the lowering effect of C22:6*n*-3 was only significant in subjects with a lower level of UA. In conclusion, C22:6*n*-3 interacts with UA to modulate glucose metabolism.

## 1. Introduction

Type 2 diabetes mellitus (T2DM) is a chronic disease characterized by high blood glucose and insulin resistance. Compelling evidence has shown that marine-derived *n*-3 long chain polyunsaturated fatty acids (*n*-3 LC PUFAs) had a beneficial effect on the prevention and treatment of T2DM. Our previous case-control study in Chinese subjects found that plasma marine-derived *n*-3 PUFA (C20:5*n*-3) was associated with improved insulin resistance [1]. A randomized controlled trial in overweight Indian subjects found that marine-derived *n*-3 LC PUFAs supplementation significantly decreased fasting blood glucose, insulin resistance, low-density lipoprotein (LDL), very low density lipoprotein (VLDL), total cholesterol, and triglycerides, but increased high density lipoprotein [2]. Our meta-analysis showed that dietary fish intake or marine-derived *n*-3 PUFA supplementation can significantly decrease the risk of T2DM in the Asian population [3].

Uric acid (UA), mostly present in plasma as monoanion urate, is the terminal oxidation product of purine metabolism [4]. UA has been reported to be an independent risk factor for T2DM [5,6] and is associated with insulin resistance [7]. Both marine-derived *n*-3 LC PUFAs and UA can modulate insulin sensitivity and blood glucose by insulin signal transduction pathway involving insulin receptor substrate (IRS) [8,9]. Many previous studies have assessed the interaction between UA and prehypertention, triglyceride as well as vitamin D_3_ on chronic diseases [10,11,12]. However, the interaction between marine-derived LC *n*-3 PUFAs and UA on T2DM has not been reported. Therefore, we conducted this case-control study to assess the interaction between plasma phospholipid (PL) marine-derived LC *n*-3 PUFAs and UA on glucose metabolism and risk of T2DM in the Chinese population.

## 2. Results and Discussion

### 2.1. Demographic Characteristics and Biochemical Parameters of Subjects

Two hundred and eleven healthy subjects in the control group and 268 T2DM subjects in the case group were included in the present study. The demographic characteristics and biochemical parameters of subjects were shown in Table 1. No significant difference was observed in sex between case and control groups. Compared with the control group, the age, plasma level of glucose, triglyceride (TG) and low-density lipoprotein cholesterol (LDL-C) was significantly higher in the case group. The plasma level of UA, total cholesterol (TC) and high-density lipoprotein cholesterol (HDL-C) was significantly lower in the case group than in the control group.

**Table 1:** Demographic characteristics and biochemical parameters in case and control groups.

Parameters	Control (*n* = 211)	Case (*n* = 268)	*p*
Age (year)	43 (36, 50)	56 (48, 64)	<0.001
Sex ^a^			0.662
Male	127 (60.2%)	156 (58.2%)	
Female	84 (39.8%)	112 (41.8%)	
Glucose (mmol/L)	5.08 (4.79, 5.42)	10.31 (7.67, 13.80)	<0.001
UA (μmol/L)	310.00 (261.00, 357.00)	276.70 (233.18, 350.78)	0.008
TG (mmol/L)	1.33 (0.97, 1.91)	1.54 (1.07, 2.19)	0.011
TC (mmol/L)	5.09 (4.59, 5.77)	4.78 (4.03, 5.61)	<0.001
HDL-C (mmol/L)	1.52 (1.21, 1.80)	1.16 (1.02, 1.40)	<0.001
LDL-C (mmol/L)	2.54 (2.05, 2.94)	2.87 (2.19, 3.41)	<0.001

^a^ Data were expressed as number (percentage). Other data were expressed as median (interquartile range); UA, uric acid; TG, triglyceride; TC, total cholesterol; HDL-C, high density lipoprotein-cholesterol; LDL-C, low density lipoprotein-cholesterol.

### 2.2. Plasma PL Fatty Acids Composition in Case and Control Groups

The plasma level of PL C16:0, C16:1, C20:3*n*-6, C20:4*n*-6, C22:4*n*-6 were significantly higher, while the plasma level of PL C18:0, C18:1, and C22:6*n*-3 were significantly lower in the case group than in the control group (Table 2). No significant difference was observed in other plasma PL fatty acids level between the two groups.

**Table 2:** Plasma fatty acids composition in case and control groups.

Fatty Acids (%, *w*/*w*)	Control	Case	*p*
C16:0	27.82 (26.21, 29.05)	28.86 (25.73, 30.74)	0.029
C16:1	0.29 (0.23, 0.34)	0.32 (0.24, 0.41)	0.003
C18:0	15.12 (14.20, 15.96)	13.43 (11.95, 14.28)	<0.001
C18:1	8.56 (7.69, 9.36)	8.11 (7.36, 9.14)	<0.001
C18:2*n*-6	22.73 (21.24, 25.21)	22.50 (19.96, 24.58)	0.076
C18:3*n*-6	0.05 (0.03, 0.11)	0.06 (0.04, 0.10)	0.374
C18:3*n*-3	0.18 (0.15, 0.25)	0.21 (0.16, 0.28)	0.381
C20:0	0.30 (0.24, 0.39)	0.31 (0.26, 0.41)	0.104
C20:1	0.24 (0.19, 0.32)	0.24 (0.19, 0.32)	0.922
C20:2*n*-6	0.41 (0.36, 0.54)	0.43 (0.34, 0.57)	0.103
C20:3*n*-6	2.32 (1.96, 2.72)	2.42 (1.85, 3.03)	0.034
C20:4*n*-6	11.43 (10.18, 12.75)	12.22 (10.53, 13.70)	0.004
C20:5*n*-3	0.94 (0.65, 1.49)	1.01 (0.58, 1.87)	0.314
C22:1	1.23 (0.66, 1.71)	0.97 (0.74, 1.23)	0.138
C22:2*n*-6	0.09 (0.04, 0.35)	0.12 (0.05, 0.41)	0.472
C22:4*n*-6	0.30 (0.25, 0.37)	0.37 (0.29, 0.55)	<0.001
C22:5*n*-3	1.15 (0.90, 1.51)	1.21 (0.89, 1.46)	0.756
C22:6*n*-3	5.21 (4.75, 5.85)	5.09 (4.08, 6.12)	0.001

Data were expressed as median (interquartile range).

### 2.3. Correlation between Plasma PL Marine-Derived n-3 LC PUFAs and Biochemical Parameters of Subjects

**Figure 1:**
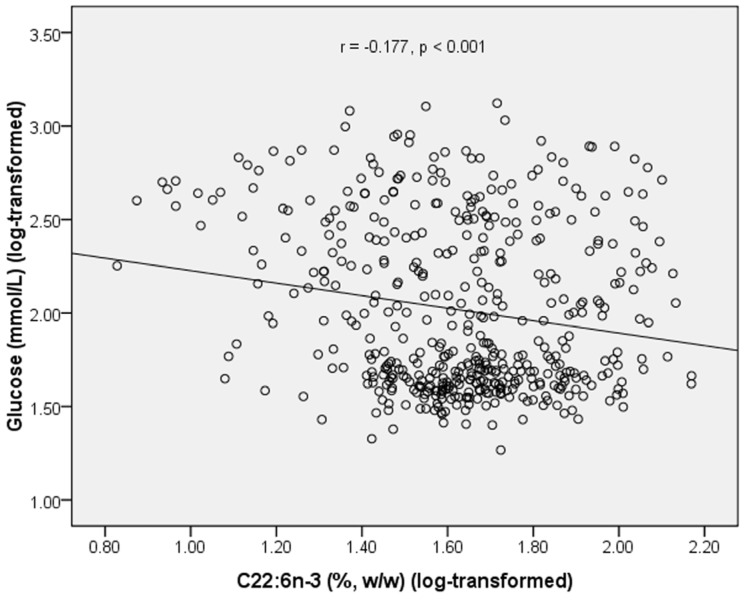
Correlation between plasma C22:6*n*-3 and fasting glucose in all subjects.

In 479 subjects, plasma PL C22:6*n*-3 was negatively correlated with glucose level (*r* = −0.177, *p* < 0.001) (Figure 1). Plasma PL C20:5*n*-3 was positively correlated with HDL-C level (*r* = 0.097, *p* = 0.036) (Table 3). Plasma level of UA was positively correlated with TG (*r* = 0.320, *p* < 0.001) and TC (*r* = 0.156, *p* < 0.001), and negatively correlated with glucose (*r* = −0.239, *p* < 0.001) and HDL-C (*r* = −0.105, *p* = 0.023) (Table 3).

**Table 3:** Pearson’s correlation coefficient between plasma marine-derived *n*-3 long chain polyunsaturated fatty acids (LC PUFAs) and biochemical parameters in all subjects.

	C20:5*n*-3	C22:5*n*-3	C22:6*n*-3	Glucose	UA	TG	TC	HDL-C	LDL-C
C20:5*n*-3	1	0.187 ***	0.380 ***	−0.032	0.082	0.044	0.048	0.097 *	0.037
C22:5*n*-3	0.187 ***	1	0.187 ***	0.006	−0.013	−0.084	−0.030	−0.040	0.037
C22:6*n*-3	0.380 ***	0.187 ***	1	−0.177 ***	0.018	0.048	0.084	0.029	0.024
Glucose	−0.032	0.006	−0.177 ***	1	−0.239 ***	0.074	−0.121 **	−0.262 ***	0.153 ***
UA	0.082	−0.013	0.018	−0.239 ***	1	0.320 ***	0.156 ***	−0.105 *	0.040
TG	0.044	−0.084	0.048	0.074	0.320 ***	1	0.349 ***	−0.284 ***	0.278 ***
TC	0.048	−0.030	0.084	−0.121 **	0.156 ***	0.349 ***	1	0.357 ***	0.715 ***
HDL-C	0.097*	−0.040	0.029	−0.262 ***	−0.105 *	−0.284 ***	0.357 ***	1	0.072
LDL-C	0.037	0.037	0.024	0.153 ***	0.040	0.278	0.715 ***	0.072	1

Data were log (e)-transformed before analysis. * *p* < 0.05; ** *p* < 0.01; *** *p* < 0.001; UA, uric acid; TG, triglyceride; TC, total cholesterol; HDL-C, high density lipoprotein-cholesterol; LDL-C, low density lipoprotein-cholesterol.

**Figure 2:**
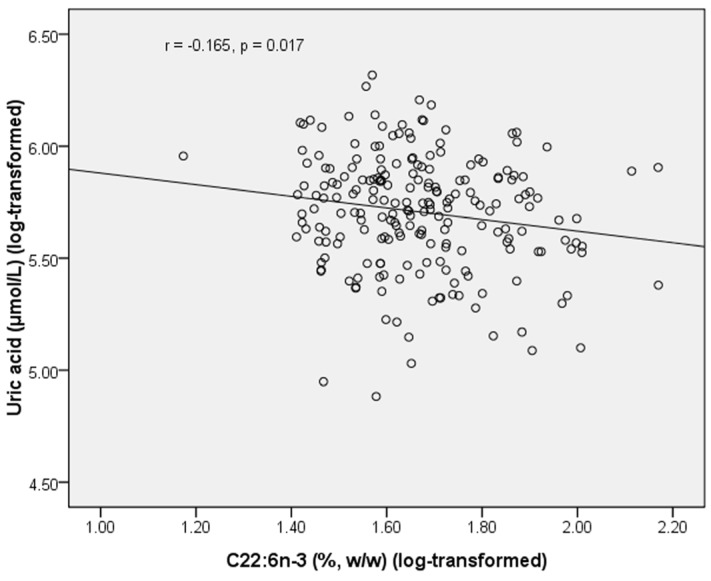
Correlation between plasma C22:6*n*-3 and uric acid in healthy subjects.

**Figure 3:**
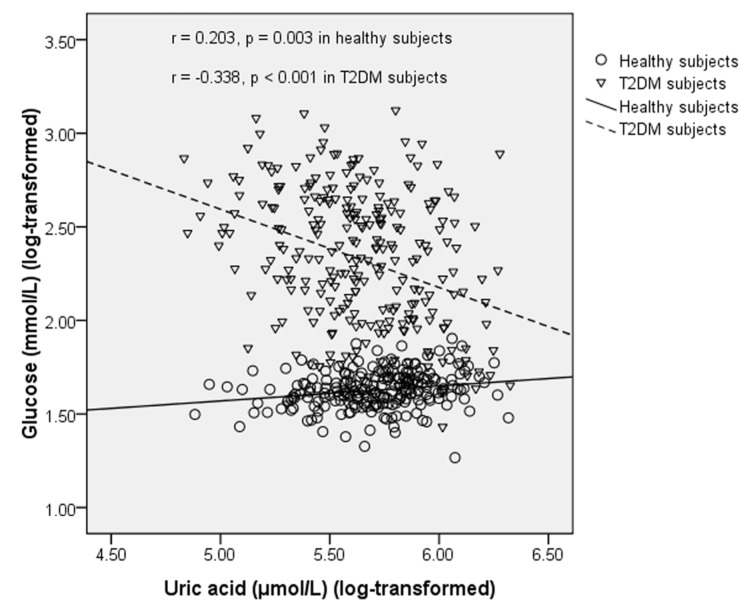
Correlation between uric acid and fasting blood glucose in healthy subjects and T2DM subjects.

We also analyzed the correlation between plasma PL *n*-3 LC PUFAs and biochemical parameters in healthy subjects and T2DM subjects separately (Table 4 and Table 5). In healthy subjects, PL C22:6*n*-3 was negatively correlated with uric acid (*r* = −0.165, *p* =0.017) (Figure 2). In T2DM subjects, PL C20:5*n*-3 was negatively correlated with glucose (*r* = −0.158, *p* = 0.011), and positively correlated with UA (*r* = 0.136, *p* = 0.029) and HDL-C (*r* = 0.131, *p* = 0.036). In T2DM subjects, C22:5*n*-3 was negatively correlated with UA (*r* = −0.124, *p* = 0.044). UA was positively correlated with glucose in healthy subjects (*r* = 0.203, *p* = 0.003), but this correlation became negative in T2DM subjects (*r* = −0.338, *p* < 0.001) (Figure 3). UA was positively correlated with TG in both healthy subjects (*r* = 0.429, *p* < 0.001) and T2DM subjects (*r* = 0.278, *p* < 0.001). UA was positively correlated with TC in both healthy subjects (*r* = 0.168, *p* = 0.015) and T2DM subjects (*r* = 0.122, *p* = 0.049). UA was negatively correlated with HDL-C (*r* = −0.279, *p* < 0.001) and positively correlated with LDL-C (*r* = 0.168, *p* = 0.014) in healthy subjects but these correlations became non-significant in T2DM subjects.

**Table 4:** Pearson’s correlation coefficient between plasma marine-derived *n*-3 long chain polyunsaturated fatty acids (LC PUFAs) and biochemical parameters in healthy subjects.

	C20:5*n*-3	C22:5*n*-3	C22:6*n*-3	GLU	UA	TG	TC	HDL-C	LDL-C
C20:5*n*-3	1	0.097	0.111	0.078	0.01	−0.057	0.121	0.114	0.039
C22:5*n*-3	0.097	1	0.016	−0.014	0.137	−0.057	0.001	−0.031	0.058
C22:6*n*-3	0.111	0.016	1	0.01	−0.165 *	−0.016	0.124	−0.032	0.098
GLU	0.078	−0.014	0.01	1	0.203 **	0.13	0.004	−0.012	0.011
UA	0.01	0.137	−0.165 *	0.203 **	1	0.429 ***	0.168 *	−0.279 ***	0.168 *
TG	−0.057	−0.057	−0.016	0.13	0.429 ***	1	0.299 ***	−0.491 ***	0.181 **
TC	0.121	0.001	0.124	0.004	0.168 *	0.299 ***	1	0.169 *	0.841 ***
HDL-C	0.114	−0.031	−0.032	−0.012	−0.279 ***	−0.491 ***	0.169 *	1	0.009
LDL-C	0.039	0.058	0.098	0.011	0.168*	0.181 **	0.841 ***	0.009	1

Data were log (e)-transformed before analysis. * *p* < 0.05; ** *p* < 0.01; *** *p* < 0.001; GLU, glucose; UA, uric acid; TG, triglyceride; TC, total cholesterol; HDL-C, high density lipoprotein-cholesterol; LDL-C, low density lipoprotein-cholesterol.

**Table 5:** Pearson’s correlation coefficient between plasma marine-derived *n*-3 long chain polyunsaturated fatty acids (LC PUFAs) and biochemical parameters in Type 2 diabetes mellitus subjects.

	C20:5*n*-3	C22:5*n*-3	C22:6*n*-3	GLU	UA	TG	TC	HDL-C	LDL-C
C20:5*n*-3	1	0.256 ***	0.526 ***	−0.158 *	0.136 *	0.115	0.027	0.131 *	0.026
C22:5*n*-3	0.256 ***	1	0.284 ***	0.042	−0.124 *	−0.105	−0.055	−0.066	0.027
C22:6*n*-3	0.526 ***	0.284 ***	1	−0.117	0.066	0.112	0.038	−0.027	0.032
GLU	−0.158 *	0.042	−0.117	1	−0.338 ***	−0.068	0.054	0.064	0.063
UA	0.136 *	−0.124 *	0.066	−0.338 ***	1	0.278 ***	0.122 *	−0.083	0.006
TG	0.115	−0.105	0.112	−0.068	0.278 ***	1	0.444 ***	−0.054	0.327 ***
TC	0.027	−0.055	0.038	0.054	0.122 *	0.444 ***	1	0.405 ***	0.749 ***
HDL-C	0.131 *	−0.066	−0.027	0.064	−0.083	−0.054	0.405 ***	1	0.224 ***
LDL-C	0.026	0.027	0.032	0.063	0.006	0.327 ***	0.749 ***	0.224 ***	1

Data were log (e)-transformed before analysis. * *p* < 0.05; ** *p* < 0.01; *** *p* < 0.001; GLU, glucose; UA, uric acid; TG, triglyceride; TC, total cholesterol; HDL-C, high density lipoprotein-cholesterol; LDLC, low density lipoprotein-cholesterol.

### 2.4. Interaction between PL Marine-Derived n-3 LC PUFAs and UA on Fasting Plasma Glucose

**Figure 4:**
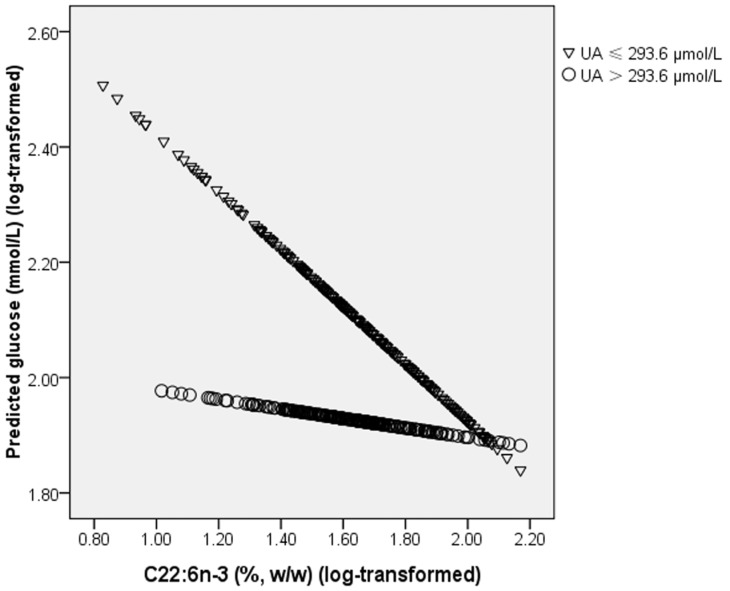
Interaction between plasma C22:6*n*-3 and uric acid on fasting blood glucose. *p* for interaction = 0.016; after adjusting for age sex, *p* for interaction = 0.005.

A significant interaction was observed between PL C22:6*n*-3 and UA on glucose levels (*p* for interaction = 0.013, adjusted for age and sex) (Table 6): higher plasma PL C22:6*n*-3 percentage was associated with a lower glucose level only when subjects had a lower level of UA, and the median (interquartile range) of glucose for increasing quartiles of PL C22:6*n*-3 was 12.02 (6.09, 14.42), 7.68 (4.99, 13.18), 6.02 (5.01, 11.60) and 7.28 (5.28, 9.65), respectively (*p* for trend = 0.002, adjusted for age and sex). When PL C22:6*n*-3 was included in the multiple linear regression model as a continuous variable, the interaction between PL C22:6*n*-3 and UA on glucose was still significant (*p* for interaction = 0.016; after adjusted for age and sex, p for interaction = 0.005) (Figure 4). After adjusting for multiple comparisons, this interaction still remained significant.

**Table 6:** Interaction between plasma marine-derived *n*-3 long chain polyunsaturated fatty acids (LC PUFAs) and uric acid (UA) on fasting glucose level (adjusted for age and sex).

Fatty Acids	Uric Acid (UA)		
	≤293.6 μmol/L	>293.6 μmol/L	*p* for Interaction
C20:5*n*-3			0.089
1st quartile	9.51 (5.28, 14.65)	6.02 (5.10, 9.20)	
2nd quartile	7.28 (5.13, 12.19)	5.47 (4.83, 8,70)	
3rd quartile	8.79 (5.10, 11.80)	5.62 (5.09, 8.18)	
4th quartile	7.36 (4.94, 10.96)	5.97 (5.28, 10.89)	
*p* for trend	0.011	0.879	
C22:5*n*-3			0.722
1st quartile	8.56 (5.16, 12.79)	5.92 (5.21, 8.67)	
2nd quartile	5.77 (4.91, 11.21)	5.91 (5.01, 8.47)	
3rd quartile	8.37 (5.12, 14.15)	6.30 (5.13, 11.22)	
4th quartile	9.68 (5.36, 13.09)	5.60 (5.02, 7.41)	
*p* for trend	0.627	0.789	
C22:6*n*-3			0.013
1st quartile	12.02 (6.09, 14.42)	7.27 (5.50, 10.92)	
2nd quartile	7.68 (4.99, 13.18)	5.37 (4.87, 6.80)	
3rd quartile	6.02 (5.01, 11.60)	5.70 (5.15, 7.85)	
4th quartile	7.28 (5.28, 9.65)	5.85 (5.13, 10.85)	
*p* for trend	0.002	0.981	

Data were expressed as median (interquartile range).

### 2.5. Association between Plasma PL Marine-Derived n-3 LC PUFAs, UA and the Risk of T2DM

Higher plasma PL C22:6*n*-3 was associated with a lower risk of T2DM, and the odds ratio (OR) was 0.32 (95% CI, 0.12 to 0.80; *p* = 0.016, adjusted for age and sex) (Table 7). No significant association was observed between PL C20:5*n*-3, PL C22:5*n*-3 or UA and the risk of T2DM.

We also assessed the interaction between plasma PL *n*-3 LC PUFAs and UA on the risk of T2DM (Table 8). A significant interaction was observed between PL C22:5*n*-3 and UA on the risk of T2DM (*p* for interaction = 0.027, adjusted for age and sex). However, after adjusting for multiple comparisons this interaction became non-significant. No significant interaction was observed when PL *n*-3 LC PUFAs was included in the logistic regression model as continuous variables.

**Table 7:** Association between plasma marine-derived *n*-3 long chain polyunsaturated fatty acids (LC PUFAs) and the risk of Type 2 diabetes mellitus (adjusted for age and sex).

Fatty Acids	OR	95% CI	*p*
C20:5*n*-3	0.98	(0.75, 1.27)	0.859
C22:5*n*-3	0.87	(0.49, 1.54)	0.623
C22:6*n*-3	0.32	(0.12, 0.80)	0.016

OR, odds ratio; CI, confidence interval.

**Table 8:** Interaction between plasma marine-derived *n*-3 long chain polyunsaturated fatty acids (LC PUFAs) and uric acid (UA) on the risk of Type 2 diabetes mellitus (adjusted for age and sex).

Fatty Acids	Uric Acid (UA)		
	≤293.6 μmol/L	>293.6 μmol/L	*p* for Interaction
C20:5*n*-3			0.986
1st quartile	1	1	
2nd quartile	0.47 (0.19, 1.17)	0.43 (0.18, 1.02)	
3rd quartile	0.51 (0.20, 1.30)	0.51 (0.22, 1.19)	
4th quartile	0.67 (0.26, 1.72)	0.91 (0.39, 2.11)	
*p* for trend	0.465	0.942	
C22:5*n*-3			0.027
1st quartile	1	1	
2nd quartile	0.60 (0.25, 1.43)	1.04 (0.45, 2.40)	
3rd quartile	1.32 (0.53, 3.29)	1.77 (0.77, 4.06)	
4th quartile	1.49 (0.59, 3.72)	0.51 (0.22, 1.20)	
*p* for trend	0.189	0.299	
C22:6*n*-3			0.372
1st quartile	1	1	
2nd quartile	0.37 (0.14, 0.98) *	0.19 (0.08, 0.46) ***	
3rd quartile	0.23 (0.09, 0.61) **	0.41 (0.18, 0.95) *	
4th quartile	0.53 (0.20, 1.42)	0.57 (0.24, 1.33)	
*p* for trend	0.166	0.580	

Data were expressed as odds ratio (OR) (95% confidence interval). * *p* < 0.05; ** *p* < 0.01; *** *p* < 0.001.

### 2.6. Discussion

In the present case-control study, we assessed the association between plasma PL fatty acid composition and blood glucose and lipid metabolism in Chinese Hans. We also assessed the interaction between plasma PL marine-derived *n*-3 LC PUFAs and UA on glucose metabolism and the risk of T2DM. We found that higher plasma PL C22:6*n*-3 percentage was associated with a lower risk of T2DM. UA showed positive correlation with fasting glucose levels in healthy subjects, but the correlation became negative in T2DM subjects. A significant interaction was observed between PL C22:6*n*-3 and UA on fasting glucose levels, and this interaction has not been reported by previous studies.

In the present study, plasma PL C22:6*n*-3 was significantly lower in the case group than in the control group, and higher PL C22:6*n*-3 was associated with a lower risk of T2DM. This result was consistent with our previous case-control study in Chinese subjects [1]. Our previous meta-analysis also found that dietary fish intake or marine-derived *n*-3 PUFA supplementation can significantly decrease the risk of T2DM in Asian populations [3]. The negative association between PL C22:6*n*-3 and risk of T2DM may be attributed to its beneficial effect on glucose and UA metabolism. T2DM is characterized by high blood glucose and insulin resistance. In the present study, we found that plasma PL C22:6*n*-3 was negatively correlated with fasting glucose level. A negative correlation between plasma PL C22:6*n*-3 and UA level was also identified in this study. Previous studies have found that UA is an independent risk factor for T2DM [5,6]. In the present study, we also found that UA was positively correlated with TG and TC in both healthy subjects and T2DM subjects; UA was positively correlated with fasting glucose, but negatively correlated with HDL-C in healthy subjects. HDL-C had a protective role against insulin resistance [13,14]. High TG level has been reported to be associated with insulin resistance [15,16]. Therefore, results in the present study also indicate the adverse effect of UA on T2DM.

Our result in healthy subjects showed that UA was positively correlated with blood glucose level. This may be attributed to the increasing effect of UA on insulin resistance [17]. Previous studies also found a negative association between UA and insulin secretion by β-cell in nondiabetic subjects [18]. This may also help explain the positive association between UA and blood glucose. However, the increasing effect of UA on the risk of T2DM observed in previous cohort studies [5,19] did not recur in the present study. On the contrary, UA was significantly lower in the case group than in the control group. This may be attributed to the osmotic diuresis caused by high blood glucose levels in T2DM, that is, the osmotic diuresis in T2DM increased the clearance rate of UA and thus lowered UA levels. In the present study, we indeed observed that UA was negatively correlated with glucose in T2DM subjects. Another reason may be that high UA levels increased the function of β cell and thus lowered blood glucose. One previous study in T2DM subjects found that UA could increase glucose disposition indices DI30 and DI120 (indices used to assess β-cell function, which combined insulin secretion and insulin resistance together) by increasing insulin secretion, although it also increased insulin resistance [20]. This may be regarded as a feedback mechanism of the human body to reduce the adverse effects of high blood glucose. However, this increased insulin secretion may accelerate the decay of β-cell function [20]. The opposite effect of UA on insulin secretion by β-cell in nondiabetic subjects and T2DM subjects observed in previous studies [18,20] may help explain why UA was positively correlated with glucose in healthy subjects but negatively correlated with glucose in T2DM subjects and why UA was lower in T2DM subjects than in healthy subjects.

Previous studies have reported the interaction between UA and prehypertension, triglyceride as well as vitamin D_3_ on chronic diseases [10,11,12]. However, the interaction between marine-derived LC *n*-3 PUFAs on chronic diseases has not been reported. In the present study, we first identified an interaction between plasma PL C22:6*n*-3 and UA on fasting glucose levels: the lowering effect of C22:6*n*-3 on blood glucose was only observed in subjects with a lower level of UA. Insulin receptor substrate 1/2 (IRS-1/2) plays an important role in insulin signal transduction [21]. Tyrosine phosphorylation of IRS leads to Src homology 2 (SH2) domain proteins binding to IRS, activates serine/threonine-specific protein kinase (AKT) and modulates glucose level in sequence [22]. A previous study in mice found that C22:6*n*-3 could significantly increase the expression of IRS-1 and IRS-2 in adipose tissue and liver [8]. This can help explain the beneficial effect of C22:6*n*-3 on glucose metabolism. One previous study found that high UA levels significantly increased serine phosphorylation of IRS-1 in mouse liver, muscle, and adipose tissue [9]. Serine phosphorylation of IRS-1 can decrease its threonine phosphorylation, and thus block the insulin signal transduction [23]. Therefore, when subjects had a higher UA level, the increased serine phosphorylation of IRS-1 counteracted the increasing effect of C22:6*n*-3 on IRS-1 expression. This can help to explain why the lowering effect of C22:6*n*-3 on glucose levels was only significant in subjects with a lower level of UA but not in subjects with a higher level of UA.

Interestingly, in the present study, the association of C20:5*n*-3 and C22:5*n*-3 with fasting glucose and the risk of T2DM, and the interaction between the two marine-derived LC PUFAs with UA on fasting glucose and the risk of T2DM were not significant. As mentioned in the paragraph, the effect of C22:6*n*-3 and UA and their interaction on glucose may be attributed to their modulating effect on insulin signal transduction pathway involving IRS-1 and IRS-2. However, previous study in rats found that C20:5*n*-3 supplementation also exhibited anti-hyperglycemic effects by increased insulin secretion, glycogen synthesis, and expression of IRS-1 [24]. The increasing effect of C20:5*n*-3 on expression of IRS-1 was also observed in a study in hepatoma cells [25]. These results seem inconsistent with our study. Two reasons may exist for the different results observed for the three marine-derived LC PUFAs in the present study. On one hand, C22:6*n*-3 is the major *n*-3 PUFA in plasma phospholipids, and the content of C22:6*n*-3 was more than four times of C20:5*n*-3 or C22:5*n*-3 in the present study. Therefore, C22:6*n*-3 in plasma phospholipids may have a greater influence on glucose than C20:5*n*-3 and C22:5*n*-3, and thus the association of C20:5*n*-3 and C22:5*n*-3 with glucose may be hidden by the variation of glucose between subjects caused by C22:6*n*-3. On the other hand, C20:5*n*-3 can be metabolized to C22:5*n*-3 and then to C22:6*n*-3 *in vivo* [26]. Although supplementation of C20:5*n*-3 can increase the expression of IRS-1 and thus exhibit anti-hyperglycemic effect, whether the beneficial effect on glucose control was induced by C20:5*n*-3 directly or by C22:6*n*-3 synthesized from C20:5*n*-3 is still unknown. Therefore, if the modulating effect of C20:5*n*-3 or C22:5*n*-3 supplementation on glucose was attributed to C22:6*n*-3 synthesized from them, the association of C20:5*n*-3 and C22:5*n*-3 with fasting glucose and the risk of T2DM, and the interaction between the two marine-derived LC PUFAs with UA on fasting glucose and the risk of T2DM may become non-significant, because C22:6*n*-3 in plasma can also be ingested from food directly.

**Figure 5:**
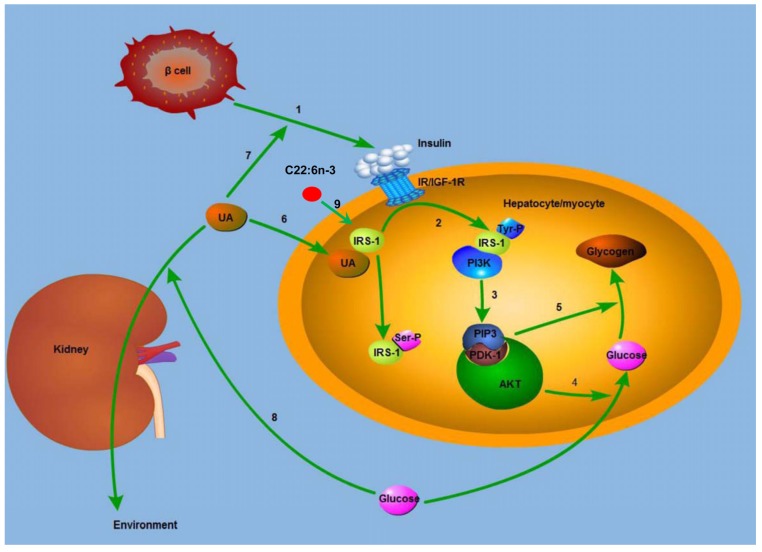
Mechanism for the modulating effect of C22:6*n*-3 and uric acid on glucose metabolism. IRS-1, insulin receptor substrate 1; IR, insulin receptor; IGF-1R, insulin growth factor 1 receptor; Tyr, tyrosine; Ser, serine; PI3K, phosphatidylinositol 3-kinase; PIP3, phosphatidylinositol 3,4,5-trisphosphate; PDK1, 3-phosphatidylinositol-dependent kinase; AKT, protein kinase B; UA, uric acid. (**1**) β cell secreted insulin, and insulin combined with IR or IGF-1R; (**2**) the complex of insulin and IR lead to the Tyr-phosphorylation of IRS-1, and the Tyr-phosphorylated IRS-1 binds to PI3K; (**3**) the complex of Tyr-phosphorylated IRS-1 and PI3K generated PIP3, which recruited AKT, and AKT is activated by PDK1; (**4**) activated AKT increased the uptake of glucose; (**5**) AKT increased the synthesis of glycogen; (**6**) UA leads to the Ser-phosphorylation of IRS-1, and thus blocked the insulin signal pathway; (**7**) UA increases the secretion of insulin by β cell in T2DM subjects, and thus lowered blood glucose; (**8**) high blood glucose leads to osmotic diuresis, increases the clearance rate of UA, and decreases plasma UA level in sequence; (**9**) C22:6*n*-3 increases the expression of IRS-1, and in this way lowers blood glucose.

The present study had several limitations. T2DM subjects may take some medications to treat hyperglycemia, hyperlipidemia, or hyperuricemia. The use of these medications in T2DM subjects may be a potential confounding factor for our results. However, information of medications use was not available in the present study, which may bias our final results. Well-designed randomized controlled trials are still needed in the future, on one hand to exclude the influence of medications use on the results, and on the other hand to see whether supplementation of C22:6*n*-3 showed different effect on glucose control in subjects with different UA level. In addition, subjects in the case group were significantly older than those in the control group, and age may bias our results. Therefore, we adjusted our primary results, the interaction between marine-derived *n*-3 LC PUFAs and UA on glucose and risk of T2DM and the association of marine-derived *n*-3 LC PUFAs and UA with risk of T2DM, for age in a multivariate linear regression model and a logistic regression model. However, after adjusting for age and sex, the interaction between marine-derived *n*-3 LC PUFAs and UA on glucose and the association between C22:6*n*-3 and risk of T2DM still remained significant, indicating that these significant results were independent of age and sex.

The potential mechanism for the modulating effect of C22:6*n*-3 and UA on glucose metabolism is shown in Figure 5.

## 3. Experimental Section

### 3.1. Subjects

Two hundred and sixty-eight subjects with T2DM were recruited from Shaoxing Hospital, Shaoxing, China. T2DM was identified if subjects had a fasting glucose level ≥7 mmol/L or had been previously diagnosed with T2DM. Two hundred and eleven healthy subjects from Zhejiang Hospital, Hangzhou, China, with a fasting glucose level <7 mmol/L and without history of diabetes or chronic diseases, such as hypertension and metabolic syndrome, were included in the control group.

The study protocol was approved by the Ethics Committee, College of Biosystem Engineering and Food Science, Zhejiang University, China on February 28, 2013, and the ethical approval code was 2013011. Written consent was obtained from all subjects prior to participation in the study.

### 3.2. Laboratory Analysis

Overnight fasting venous blood samples were collected with 21-gauge needles in the morning. Plasma samples were prepared after blood collection as soon as possible, aliquoted into separated tubes and stored at −80 °C until analysis. Method for fasting glucose detection has been described in our previous study [27]. Plasma UA and lipids were determined by an autoanalyzer (Olympus AU2700, Tokyo, Japan). Total lipid content of plasma was extracted by chloroform/methanol (1:1), the PL fractions were separated by thin layer chromatography, and the fatty acid methyl esters were prepared and separated by gas-liquid chromatography [28].

### 3.3. Statistical Analysis

Categorical variables were tested by Pearson’s chi-square test. Normal distribution tests were conducted for continuous variables. Data that were not normally distributed were log (e)-transformed before analysis. All continuous variables were expressed as median (interquartile range) because data of most continuous variables were not normally distributed. One-way analysis of variance (ANOVA) analysis was used to compare means. Pearson’s correlation coefficient was used to test the correlation between biochemical parameters and fatty acids. A multivariate linear regression model was used to test the interaction between plasma PL *n*-3 LC PUFAs and UA on fasting glucose levels, adjusted for age and sex. A logistic regression model was used to calculate the odds ratios (ORs) of T2DM with respect to per unit increase of plasma PL *n*-3 LC PUFAs and UA or the interaction between PL *n*-3 LC-PUFAs and UA, adjusted for age and sex. A *p* value < 0.05 was considered statistically significant. We also adjusted our primary results, interaction between 3 plasma PL marine-derived *n*-3 LC PUFAs and UA on fasting glucose or the risk of T2DM, for multiple comparisons (Bonferroni correction). Therefore, a *p* value < 0.017 (0.05/3) was considered significant after multiple comparisons adjustment. All data analyses were conducted by SPSS 16.0 (SPSS, Inc., Chicago, IL, USA).

## 4. Conclusions

C22:6*n*-3 interacts with UA to modulate glucose metabolism. C22:6*n*-3 supplementation together with UA-lowering medicine may lead to better control of blood glucose levels in subjects with high UA levels.
